# Analytical Performance and Validation of a Reliable Method Based on Graphite Furnace Atomic Absorption Spectrometry for the Determination of Gold Nanoparticles in Biological Tissues

**DOI:** 10.3390/nano11123370

**Published:** 2021-12-12

**Authors:** Oana Cadar, Teodora Mocan, Cecilia Roman, Marin Senila

**Affiliations:** 1 INCDO-INOE 2000, Research Institute for Analytical Instrumentation, RO-400296 Cluj-Napoca, Romania; oana.cadar@icia.ro (O.C.); cici_roman@yahoo.com (C.R.); 2 Physiology Department, “Iuliu Hatieganu” University of Medicine and Pharmacy, RO-400006 Cluj-Napoca, Romania; teodora.mocan@umfcluj.ro; 3 Nanomedicine Department, Regional Institute of Gastroenterology and Hepatology, RO-400158 Cluj-Napoca, Romania

**Keywords:** gold nanoparticles, GF-AAS, validation, biological tissue

## Abstract

Gold nanoparticles (AuNPs) have a wide-ranging application and are widespread in samples with complex matrices; thus, efficient analytical procedures are necessary to identify and characterize this analyte. A sensitive analytical method for determination of AuNPs content in biological tissues, based on microwave-assisted acid wet digestion and graphite furnace atomic absorption spectrometry (GFAAS) validated in accordance with the requirements of Eurachem guideline and ISO 17025 standard, is presented in this study. The digestion procedure was optimized, and the figures of merit such as selectivity, limit of detection (0.43 µg L^−1^), limit of quantification (1.29 µg L^−1^, corresponding to 12.9 µg kg^−1^ in tissue sample, considering the digestion), working range, linearity, repeatability ((RSD_r_ 4.15%), intermediate precision (RSD_R_ 8.07%), recovery in accuracy study (97%), were methodically evaluated. The measurement uncertainty was assessed considering the main sources of uncertainties and the calculated relative expanded uncertainty (k = 2) was 12.5%. The method was applied for the determination of AuNPs in six biological tissues (liver, small intestine, heart, lungs, brain and kidneys) and the found concentrations were generally at low levels, close or lower than LOQ.

## 1. Introduction

Nanomaterials play an important role in our day-to-day life due to their wide-ranging applications [1]. Among the different metallic nanoparticles, due to their unique optical, sensing, and biochemical characteristics, the gold nanoparticles (AuNPs) have been progressively recognized as one of the most promising materials in biomedical sciences, including gene and drug delivery, imaging, molecular diagnostics and therapies [2]. However, several concerns and arguments on the safety of nanomaterials and their use in consumers good have been raised [3]. Owing to their very small sizes, the ingested nanomaterials may be retained in some tissues, causing toxicity. The biological safety of nanomaterials is closely related to their concentration and particle size to be absorbed, distribution and metabolism in the body. Furthermore, the translational research of AuNPs in nanomedicine can be fully accomplished only after acquiring an almost entire understanding of their potential risks and hazards [4].

Nowadays, the development of reliable and robust analytical methods that permit rapid, simple and cost-effective information about the size and metal concentration of NPs is a topic of increasing interest [1,4]. The existing analytical methods for the AuNPs characterization can be divided into two classes, i.e., (1) methods without AuNPs digestion and (2) methods with a previous step to obtain dissolved Au species into solution [5]. Consequently, light scattering, mass spectrometry, colorimetry, UV-Vis spectroscopy and imaging techniques such as scanning electron microscopy (SEM) and transmission electron microscopy (TEM) were employed for determination of the total number of nanoparticles in dispersions, without digestion [6,7,8]. However, the AuNPs dispersions with particle size in the range of 1–100 nm are difficult to be quantitatively analyzed without a digestion step [9]. Additionally, quantitative methods that require a digestion of AuNPs are mainly electrochemical, mass spectrometry and optical spectrometry.

Two main groups of analytical methods based on atomic optical spectrometry are used for AuNPs determination, the main differences between these techniques consisting in the type of atomization source, which leads in variances in detection limits. In the first group of analytical techniques, the emission spectra of analyte obtained in an inductively coupled plasma (ICP) is registered at specific wavelengths [10,11]. The second group of optical atomic spectrometric techniques is based on the absorption of radiation at a specific wavelength by the atoms of analyte. In this case, the atoms can be obtained by electrothermal evaporation in a graphite furnace (GFAAS) or flame (FAAS) absorption spectrometry [12,13]. Among the optical spectrometry techniques, GFAAS provides the lower detection limits which represent an advantage for the determination of AuNPs in biological tissues where is expected to have low Au concentrations. Another important advantage of GF-AAS is that it requires low sample volume, and moreover, in GFAAS the complex organic matrices can be eliminated prior to atomization of analyte [14], so this technique can be considered as a powerful tool for the determination of AuNPs analysis in complex matrices.

In several studies, GFAAS was used for distinguishing between dissolved forms and NPs of Au in real samples. It was reported that higher temperatures are required for the atomization of NPs forms comparing with those for dissolved forms. However, when using conventional GFAAS for the determination of AuNPs in biological tissues, it is recommended to include a sample preparation step in order to decompose the sample matrix and to transform AuNPs into ionic form. This step is essential in obtaining quantitative recovery of the analyte, and thus accurate results [15,16,17,18,19,20].

Comparing with other heating techniques for wet acid samples digestion, the closed microwave-assisted wet digestion/decomposition-based technique is widely recognized to have several advantages including fast heating, low risk of sample contamination and low amounts of oxidant agents (mineral acids). Usually, the AuNPs are decomposed by aqua regia, but the matrix composition should be also considered when the acids are chosen for the digestion step development [21].

Although the amount of AuNPs in different tissues is an important consideration for their use in medical applications, no international standard method presenting the methodology for AuNPs determination in biological tissues is available. Consequently, in this field, there is a need of robust analytical methodologies obtained in a fully-validated process. Thus, efficient analytical approaches are necessary to identify and characterize the AuNPs in various and complex matrices for the quality control of products/materials as well as risk assessment purposes.

The requirements of the International Organization for Standardization (ISO) 17025:2017 for a testing laboratory are to demonstrate its expertise in methods of research. The laboratory shall demonstrate its capabilities to apply non-standard methods, laboratory-designed or developed methods, standard methods used outside their intended scope, or modifications of standard methods trough a validation process [22].

In this context, the aim of this study was the validation of an analytical methodology based on microwave-assisted wet digestion and GFAAS technique for determination of AuNPs in biological tissues. Since a complete digestion of the sample containing AuNPs led to the Au(III) formation [9,21], only the performance parameters for these two species were evaluated in this study. We considered that Au(I) is an intermediate species which is finally released in solution as Au(III), when using an excess of acids for digestion. The optimization of the sample volume, acidic mixture and microwave program for decomposition of the AuNPs is described. The method was characterized in terms of selectivity, limit of detection (LOD), limit of quantification (LOQ), accuracy, precision, linearity of calibration curve and uncertainty. Finally, the validated methodology was used for AuNPs determinations in tissues. The validation was performed considering the recommendations of the Cooperation for Analytical Chemistry in Europe (EURACHEM) guide [23].

## 2. Materials and Methods

### 2.1. Reagents, Standard Solutions and Certified Reference Material

Nitric acid 65% (*w*/*w*), hydrochloric acid 37% (*w*/*w*) and H_2_O_2_ 30% (*w*/*w*) of analytical grade, and single-element standard solution 1000 mg L^−1^ of Au were purchased from Merck (Darmstadt, Germany). Colloidal AuNPs with a nominal diameter of 30 nm with Au mass fraction of 45.1 mg kg^−1^ from LGC (Bury, UK) was used as quality control solution and to spike the samples. Ultrapure water (18 MΩ cm) prepared with Milli-Q system Direct Q3 (Millipore, Molsheim, France) was used throughout the experiments.

### 2.2. Methods and Instrumentation

A closed-vessel Xpert microwave system (Berghof, Eningen, Germany) was used for samples digestion. All PTFE digestion vessels were previously soaked in 10% (*v*/*v*) nitric acid solution for 48 h to avoid cross-contamination. An amount of 200 mg biological tissue sample was digested using 10.5 mL mixture of HNO_3_ 65% and HCl 37% (1:3, 1:6, 1:9, 9:1, 6:1 and 3:1, *v*/*v*) in PTFE digestion vessels, using a four-step digestion program (120 °C and 190 °C—heating, 100 °C and 25 °C—cooling) for a total digestion time of 40 min. When the vessels cooled down, the digested samples were transferred in 20 mL volumetric flasks and diluted to the mark with ultrapure water. The reagent blank was prepared by using the same volume and acid mixtures and following the procedure used to prepare the real samples. Each sample was prepared in triplicate.

A graphite furnace atomic absorption spectrometer Perkin Elmer model PinAAcle 900T (Norwalk, CT, USA) was used for determination of Au in samples. Sample aliquots of 20 μL were directly injected into the graphite tube, and then, a volume of 5 μL of chemical modifier (containing 0.005 mg Pd + 0.003 mg Mg(NO_3_)_2_) was added. The matrix modifier was used according to the recommendation of the instrument manufacturer. The operating conditions in GFAAS are given in Table 1.

In order to check the possible influence of Au species (Au (III) and AuNPs) on the analytical signal, two seven-point external calibrations were plotted for Au (III) and AuNPs using the reagent blank and six calibration standard solutions of 1.0, 2.0, 5.0, 8.0, 12, 15 and 20 µg L^−1^. The calibration standards were prepared by auto dilution of the highest concentrated standard solutions of Au (III) and AuNPs (20 µg L^−1^) with the reagent blank, using the instrument autosampler. In order to check the calibrations, the highest concentrated standard solution from each calibration curve was measured to be in the range of ±10% from the theoretical value. The measured values were in the required range.

After microwave digestion, the samples were transferred to autosampler vials. A volume of 20 μL sample and 5 μL matrix modifier were delivered in each analysis. Three measurements were done for each sample solution on the calibration curve built using Au(III) solution.

The GF-AAS method was characterized regarding selectivity, LOD, LOQ, linearity of calibration curve, precision, accuracy and measurement uncertainty.

### 2.3. Synthesis of Nanomaterial

Synthesis of AuNPs has carried out using a modified Turkevich method. In short, HAuCl_4_ was dissolved in distilled water under heating conditions (100 °C). Stabilization through rapid citrate addition was carried out, continued by 3 h—continuous reflux under mechanical stirring conditions. Solution color shift was observed and monitored. Functionalization with immunogenic peptide was performed using a first step of DL-dithiotreitol (DTT) mixing, followed by AuNPs addition under continuous stirring conditions for 1.5 h. Centrifugation of the obtained GNP-peptide solution was performed (15,000 rpm/40 min), followed by the pellet redispersion in ultrapure water.

### 2.4. Animal Autotransplant Model

Peritoneal macrophages of 12 *Mus musculus* mice were isolated by peritoneal injection and washing with saline solutions (phosphate buffered saline, PBS with 3% fetal bovine serum, FBS), followed by aspiration of peritoneal liquid. Separate primary cultures were performed using an already validated protocol [24]. Next, the control group samples of macrophages culture were treated in vitro with PBS, whilst the rest of the test samples received a different concentration of nanomaterial through in vitro exposure, namely 12.5 µg/mL (*n* = 3). Seventy-two hours after exposure, each primary cell sample was readministered to the animal where it was initially extracted from. Readministration was carried out by cell resuspension in PBS and intraperitoneal injection. One week after autotransplant, animals were euthanized, and tissues and organs were collected. Animal protocol has sought and obtained all legal approval (267/12.07.2021 by National Sanitary Veterinary and Food Safety Authority of Romania).

## 3. Results and Discussion

### 3.1. Optimization of Microwave-Assisted Acid Wet Digestion Procedure

The quantitative extraction recovery of the target analyte in an analytical procedure is essential in order to provide consistent results. The wet acid digestion proves to be effective on both organic and inorganic substances due to its ability to destroy the sample matrix and, consequently, minimize the interferences. In this regard, the wet acid digestion of precious metals, such as Au, is a simple, rapid and low-cost procedure, but the recovery efficiency of acid extraction is highly dependent on the type of matrix, chemical solubility and concentration of analytes, etc. Some sources of incomplete digestion are rational selection and amount of acid mixtures, incomplete wetting of solid samples and occlusion of metals in the solid support [11]. The use of microwave-assisted digestion allows high temperatures and pressures, and can considerably enhance the leaching of analyte (Au), but the ratio of mineral acids used can significantly influence the extraction.

In order to ensure a complete digestion of biological samples, different mixtures of HNO_3_ 65% and HCl 37% in ratios of 1:3 (*v*/*v*), 1:6 (*v*/*v*), 1:9 (*v*/*v*), 9:1 (*v*/*v*), 6:1 (*v*/*v*) and 3:1 (*v*/*v*) were used in microwave conditions, by applying the same digestion program for the digestion of 200 mg sample. The samples resulted from the biological tissue (heart, control) fortified with AuNPs solution at a concentration of 200 µg kg^−^^1^ Au in initial samples were used to assess the Au recovery and the extraction efficiency respectively. Three replicates were performed for this experiment, with an average repeatability standard deviation of 5%. The recovery of total Au ranged between 87.1–102.7%. Since GFAAS techniques measures the total Au concentration, in our case the recovery refers to the extraction of the total Au (Au(III) and AuNPs) species from the solid biological sample into the digested liquid sample. The highest recovery being obtained for a HNO_3_:HCl ratio of 1:6 (*v*/*v*). Considering 200 mg biological sample and the Au concentration of 200 µg kg^−1^, the fortified sample contains 0.04 µg Au. Considering the redox reaction that occurs into the samples (Equations (2) and (3)), for this Au amount are necessary to react 0.013 µg HNO_3_ and 0.022 µg HCl. In all cases, the added amount of each acid was in excess (0.9–8.6 g HNO_3_ and 0.4–4.2 g HCl, respectively) in the total mixture volume of 10.5 mL. Therefore, no significant change was observed if the ratio was 1:9 (*v*/*v*) and 1:3 (*v*/*v*) or 3:1 (*v*/*v*), 6:1 (*v*/*v*) and 9:1 (*v*/*v*). However, given the highest obtained recovery, a HNO_3_:HCl ratio of 1:6 (*v*/*v*) was considered as optimum. For the quantitative leaching of Au from 200 mg biological tissue, the use of 1.5 mL of HNO_3_ 65% and 9.0 mL HCl 37% in microwave-assisted conditions was considered an appropriate digestion method, even if much lower amounts of acids are required to react with Au for its dissolution at a level of concentration similar to the fortified sample used in this study. Furthermore, a higher amount of acid mixture can provide the dissolution of possible higher Au concentrations in unknown samples, as well as the matrix sample decomposition.

The developed microwave-assisted extraction method requires small volumes of acid mixtures and amount of sample, offers quantitative recoveries under optimal conditions and short time (40 min). Though, beside the sufficient digestion of target analyte (Au), the quantitative analysis by the appropriate instrument and experimental parameters needs to be selected.

### 3.2. Performance Parameters

#### 3.2.1. Selectivity

Selectivity indicates the ability of the analytical method to suitably quantify Au in the presence of interferences. To evaluate this parameter, a digested tissue solution was spiked with 5 μg L^−1^ Au and measured by GFAAS, and the average recovery was 108.2%, within the range of 90–110%. The effect of major constituents such as Ca^2+^, Mg^2+^, Na^+^, K^+^, Cl^−^, NO_3_^−^, SO_4_^2−^ and organic carbon at concentration levels of 1000 mg L^−1^ on Au determination at a level of 5 μg L^−1^ was verified, and non-significant matrix effects on the analytical signal were observed. These obtained results prove that the method can be applied to the Au analysis even in matrices containing high concentrations of foreign ions resulted from the digestion of biological tissues.

#### 3.2.2. Working Range, Limit of Detection and Limit of Quantification 

The working range is the interval over which an analytical method provides results with a satisfactory measurement uncertainty [25]. The lower limit of the range is given by the LOQ, while the upper limit is influenced by the analytical signal of equipment. The working range was determined by statistical analysis (Snedecor’s *F* test) based on the ISO 8466-1 [26] recommendations on the extreme concentration levels of calibration standards. Homogeneity of variances (PG) was assessed as variance ratio for the extreme concentration levels of calibration standards. To evaluate this parameter, solutions with concentrations of 1.0 µg L^−1^ and 20 µg L^−1^ containing Au(III) and AuNPs species, respectively, were analyzed. 

**Figure 1:**
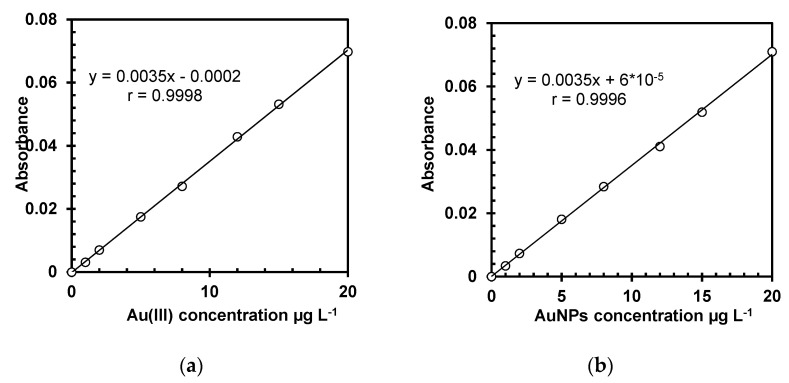
Linear calibration curves for Au analysis by GFAAS (**a**) using Au(III) solution and (**b**) using AuNPs dispersion.

The PG value for Au(III) was 3.31, while the PG value for AuNPs was 4.23, in both cases lower than the critical value of Fisher–Snedecor distribution (F_9;9;0.99_ = 5.35) showing that, from this point of view, the calibration range was chosen correctly. Subsequently, the calibration curves were built by measuring a blank solution and seven concentration levels (1.0, 2.0, 5.0, 8.0, 12, 15 and 20 µg L^−1^), and the obtained correlation coefficients were close to 1 (r = 0.9998 for calibration built using Au(III) and r = 0.9996 for calibration built using AuNPs), as presented in Figure 1, indicating a good linearity [25].

Generally, LOD is considered as the lowest concentration that can be measured consistently. There are several methods for the estimation of LOD; in this work, the LOD was calculated using the 3s_y/x_/m criterion, where s_y/x_ is the residual standard deviation of the calibration curve, y is the intercept and m is the slope of the calibration curve, according to Equation (1) [27]:

LOD = (3 s_y/x_ − y)/m (1)

LOQ is the lowest concentration of analyte that can be measured with an acceptable level of accuracy and precision. In this paper, LOQ was calculated as three times the LOD. LODs calculated for both Au(III) and AuNP species considering the characteristics of the two calibration curves over the range 0–20 µg L^−1^ are presented in Table 2. 

As shown in Table 2, there are no statistically significant differences between the characteristics of the two calibration curves (values ± standard deviations of intercepts and slopes), indicating that Au can be quantitatively measured in AuNP dispersions without any digestion. The determination of AuNPs without digestion by atomic absorption spectrometry (AAS) was previously reported by several authors [5,9], but in this study the acid digestion (HCl + HNO_3_) of biological tissue was necessary to extract the analyte into an aqueous solution from biological complex matrix for further determination. This step leads to the Au from AuNPs digestion to Au(III) form, according to the chemical reactions equations [9]:

HNO_3_ + 3HCl = 2H_2_O + Cl_2_ + NOCl (2)

Au + Cl_2_ + NOCl = AuCl_3_ + NO↑ (3)

Considering the similitude of analytical signal for the both Au species and the transformation of AuNPs into Au(III) through the acid digestion step, all the analytical determinations and evaluation of the method performance parameters were done on the calibration curve built using Au(III) solution. Values of LOD and LOQ were finally calculated in a solid sample taking into account the quantity of the sample used for digestion and the final volume of the digested sample. The LOD was 0.43 µg L^−1^ in liquid solution, while LOQ was 1.29 µg L^−1^ that corresponds to 12.9 µg kg^−1^ in the solid tissue sample.

The LOQ value was verified in terms of repeatability and recovery, by analyzing an Au solution prepared at a level of concentration close to LOQ (1.00 µg L^−1^). The RSD for repeatability (six parallel measurements) was 12%, in the imposed target of 20%, while recovery was 93%, also in the imposed target of 85–115% [13]. Lower LOD of 19.5 ng L^−1^ and 58.7 ng L^−1^ for AuNPs determination by GFAAS were reported by Garcia-Figueroa et al. [28], but calculated using *3s* criterion, based on the standard deviation of analytical response resulted from repeated blank measurements.

### 3.3. Precision and Accuracy

Repeatability and intermediate precision were assessed by the application of one way of variance as prescribed in the ISO 5725-5 standard [29]. To achieve this, six parallel determinations were carried out on samples resulted from biological tissue (heart, control) fortified with AuNPs solution, to correspond at a concentration of 200 µg kg^−^^1^ Au in initial samples. Analyses were done under the same conditions by a single analyst, during the same day (repeatability), and under the same conditions by a single analyst, but in different five days (intermediate precision or reproducibility). Table 3 summarizes the obtained results for the repeatability and intermediate precision assays. Relative standard deviation of repeatability (RSD_r_) and relative standard deviation for intermediate precision (RSD_R_) were then compared with predicted relative standard deviations (PRSD%) calculated according to Horvitz’s equation (Equation (4)) [30]:

(4) $$\mathrm{PRSD}\%=2^{\left(1-0.5\mathrm{logC}\right)}$$

where C is the analyte concentration expressed as mass fraction in extracted liquid solutions (200 µg kg^−^^1^).

The relative standard deviation of repeatability (RSD_r_) was 4.15%, while the relative standard deviation for intermediate precision (RSD_R_) was 8.07%, lower than PRSD% (14.4%), thus being considered as acceptable. Garcia et al. reported intra-day repeatability and inter-day reproducibility, expressed as relative standard deviation of 5.3% and 7.6% for AuNPs, and 2.5% and 5.3% for Au(III) respectively, for water samples [28].

The accuracy reflects the close agreement among a conventional true value or an acknowledged reference value from a reference material with the measured value [31]. In practice, the accuracy is usually estimated by measuring a reference material with a certified content of analyte in a matrix similar to the analyzed sample. However, in some cases, a certified reference material with similar matrix does not exist, and in this case the accuracy may be assessed by percentage of analyte recovered from fortified sample. In our case, known concentrations of AuNPs were added to biological tissue, and the resulted samples were analyzed using all the steps of the analytical procedure. The obtained results were compared to the value of reference material added. For an instrumental analytical method, at a concentration of analyte of hundreds ppm, a recovery within the range 85–110% is considered to be satisfactory [32]. The obtained recovery (97%) was within the acceptance criteria, and therefore, the developed method was considered accurate for the quantification of AuNPs in biological tissues.

### 3.4. Measurement Uncertainty

**Figure 2:**
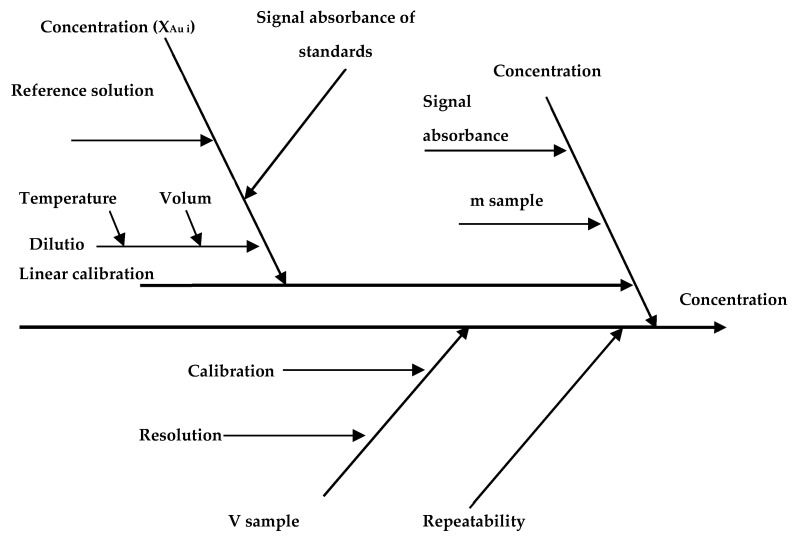
Cause and effects diagram (Ishikawa diagram) of uncertainties in the measurement of Au in biological tissue using GFAAS.

The method validation demonstrates the consistency of the analytical results, but it is not sufficient to accurately interpret and compare the obtained results [33]. In this regard, the uncertainty quantification of the AuNPs in biological tissue was estimated based on the model (y = f(x_1_, x_2_, …, x_n_)) that physically represents the quantities involved in the measurement process [34,35]. The model for Au quantification in biological tissue is described in Equation (5):

(5) $$X_{\mathrm{Au}}=\frac{X_{\mathrm{Aui}}*\mathrm{Vs}}{m\;\mathrm{sample}}\mathrm{rep}$$

where X_Au_ is the final concentration of AuNPs in biological tissue sample, X_Aui_ is the Au concentration measured by GFAAS, m sample is the sample biological tissue mass and rep is a repeatability factor included into the model to take random uncertainty components (type A) into consideration [34,35]. Considering the measurement model components, each source of uncertainty was identified, according to the Ishikawa diagram presented in Figure 2.

The identified major sources of measurement uncertainty were uncertainty of calibration reference materials, uncertainty of delivered volumes, uncertainty of measured intensities of the reference solutions and uncertainty of weighted samples and repeatability of the method. The uncertainty of delivered volumes (from volumetric flasks, pipettes) was calculated by using the manufacturer data on calibration certificates and the uncertainty associated with the use of glassware at a temperature different from that of volumetric deliveries. Uncertainty of sample weighting was calculated from data obtained from calibration certificates of analytical balance and the repeatability of weighing. The uncertainty of repeatability was estimated as standard deviation of the parallel measurements. After estimation, the sources of uncertainty were combined according to the law of propagation of uncertainties, to obtain the combined standard uncertainty (u_c_). The expanded uncertainty (U) was calculated by multiplying the combined standard uncertainty with a coverage factor (k = 2), corresponding to a 95% confidence level. Calculated from these sources, the relative expanded uncertainty (Urel) was 12.5%.

### 3.5. Application of GFAAS-Based Method to Biological Samples

The obtained results for Au determination in the harvested organs (liver, small intestine, heart, lungs, brain and kidneys) indicated that Au accumulated in low level in liver (15.3 µg kg^−1^), while there was extremely low level of Au in the other organs (below the LOQ of 12.9 µg kg^−1^). The proposed procedure demonstrated to be an outstanding method in the quantification of Au metallic nanoparticles-based compounds with complex matrices and a high content of organic matter, without the need for an additional step to reduce or to eliminate the matrix interferences.

The validated method could become a useful tool for evaluating side-effects of targeted nanomediated applications, by evaluating the limited, if existent, presence in other tissues/organs apart from the targeted tissue. However, our results are requiring the need for further studies in order to attain better in vivo compliance and exploit the enormous potential of AuNPs in cancer therapy.

## 4. Conclusions

In this study, we validated a method for the quantification of AuNPs in biological tissues based on microwave-assisted acid wet digestion and graphite furnace atomic absorption spectrometry (GF-AAS) according to the requirements of international Eurachem guideline and ISO 17025 standard. In addition, the overall measurement uncertainty was determined. A ratio of HNO_3_:HCl of 1:6 (*v*/*v*) with a total time of digestion of 40 min was found to give recoveries in the target values of 80–120%. The obtained performance parameters were in agreement with the requirements of international guidelines regarding methods validation. The validated method was tested further by analyzing real tissue samples (liver, small intestine, heart, lungs, brain and kidneys). The obtained results for Au determination in the investigated organs showed that Au accumulated in low level in liver, while in the other organs, the Au level was below LOQ. The obtained results confirmed that the validated method is appropriate for the quantitative determination of Au in biological tissues after gavage administration of AuNPs. Moreover, the digestion method can be applied to other concentration testing techniques that require dissolving AuNPs.

## Figures and Tables

**Table 1:** Operation conditions for Au determination by graphite furnace atomic absorption spectrometry (GFAAS).

GFAAS				
Signal processing: Peak area; Read time: 5 s; Sample volume: 20 µL Background correction: Zeeman-effect				
Wavelength—242.80 nm Slit—0.7 nm				
**Furnace program Pd**				
Step	Temp (°C)	Ramp (s)	Hold (s)	Ar (mL min^−1^)
Drying	110	1	30	250
Drying	130	15	30	250
Ashing	800	10	20	250
Vaporization	1800	0	5	250
Cleaning	2450	1	2	250

**Table 2:** Characteristics of the calibration curves over the range 0–20 µg L^−1^.

Species	Intercept (y)	Slope (m)	Residual Stdev. (s_y/x_)	Correlation Coeff. (r)	LOD (µg L^−1^)
Au(III)	−0.00017 ± 0.00031	0.00352 ± 0.00003	0.00057	0.9998	0.43
AuNPs	0.00006 ± 0.00036	0.00350 ± 0.00004	0.00067	0.9996	0.56

**Table 3:** Results obtained in the repeatability/intermediate precision assays the Au analysis from biological tissues by GFAAS (*n* = 6 parallel samples).

Measurements	Repeatability	Intermediate Precision
X_1_ (µg kg^−^^1^)	187	194
X_2_ (µg kg^−^^1^)	193	206
X_3_ (µg kg^−^^1^)	188	215
X_4_ (µg kg^−^^1^)	203	185
X_5_ (µg kg^−^^1^)	190	217
X_6_ (µg kg^−^^1^)	206	178
X_m_ (µg kg^−^^1^)	194	199
s (mg kg^−1^)	8.07	16.1
r/R (mg kg^−1^)	22.6	45.0
RSD_r_/RSD_R_ (%)	4.15	8.07

s—standard deviation, r—limit of repeatability (r = s × 2.8); R—limit of reproducibility (intermediate precision), RSD_r_—relative standard deviation of repeatability, RSD_R_—relative standard deviation of reproducibility (intermediate precision).

## Data Availability

Not applicable.
